# Attenuated and delayed neural activity in cortical microcircuitry of monocular processing and binocular interactions in human amblyopia

**DOI:** 10.1162/imag_a_00561

**Published:** 2025-04-30

**Authors:** Yue Wang, Chencan Qian, Yige Gao, Yulian Zhou, Xiaotong Zhang, Wen Wen, Peng Zhang

**Affiliations:** State Key Laboratory of Brain and Cognitive Science, Institute of Biophysics, Chinese Academy of Sciences, Beijing, China; University of Chinese Academy of Sciences, Beijing, China; Department of Ophthalmology & Visual Science, Eye & ENT Hospital, Shanghai Medical College, Fudan University, Shanghai, China; State Key Laboratory of Medical Neurobiology and MOE Frontiers Center for Brain Science, Institutes of Brain Science, Fudan University, Shanghai, China; Key Laboratory of Myopia, Ministry of Health, Fudan University, Shanghai, China; Shanghai Key Laboratory of Visual Impairment and Restoration, Fudan University, Shanghai, China; College of Electrical Engineering, Zhejiang University, Hangzhou, China; Second Affiliated Hospital, School of Medicine, Zhejiang University, Hangzhou, China

**Keywords:** layer fMRI, 7 Tesla, amblyopia, EEG frequency tagging, binocular interaction

## Abstract

Disruption of retinal input early in life can lead to amblyopia, a condition characterized by reduced visual acuity after optical correction. While functional abnormalities in the early visual areas have been observed in amblyopia, mesoscale deficits in cortical microcircuitry across cortical depth remain unexplored in humans. Using a combination of submillimeter 7T fMRI and EEG frequency-tagging methods, we investigated neural deficits in monocular processing and binocular interactions in human adults with unilateral amblyopia. The results revealed attenuated and delayed monocular activity in the thalamic input layers of V1, followed by imbalanced binocular suppression and weakened binocular integration in the superficial layers. These disruptions further reduced visual signal strength and processing speed. Our findings pinpoint specific neural deficits in the cortical microcircuitry associated with human amblyopia, offering valuable insights into the mesoscale mechanisms of developmental plasticity and paving the way for more effective treatments for this visual disorder.

## 1 Introduction

Amblyopia, also known as “lazy eye,” is a common neurodevelopmental disorder characterized by visual acuity loss due to early life disruptions of retinal input, such as anisometropia (unequal refractive error) or strabismus (misalignment of eyes). Amblyopia serves as an ideal model for studying how abnormal experiences shape the brain function and wiring during human development. Understanding the specific neural deficits is crucial for designing more targeted and effective therapeutic interventions. While functional abnormalities have been identified in the visual system of human amblyopes, it remains unclear about the mesoscale deficits in the cortical microcircuitry underlying monocular processing and binocular interactions.

Monocular deficits of amblyopia have been found at multiple levels of the visual hierarchy (Levi, 2020). However, for most of these findings, it remains unclear whether they reflect abnormalities in feedforward or feedback processing. For example, functional deficits of amblyopia could arise from the early visual areas and then propagate to higher cortex in feedforward processing. Alternatively, high-level deficits may also influence activity in the early visual areas through feedback modulations. In support of the deficits in feedforward processing, cytohistology (Adams et al., 2007) and high-resolution fMRI (Goodyear et al., 2002) showed a shrinkage of the amblyopic eye’s column in the primary visual cortex (V1) of early onset patients, likely due to disrupted visual input in the critical period of the development of ocular dominance columns (ODCs). However, for most human amblyopes, there was no shrinkage in ODCs or the lateral geniculate nucleus (LGN) of the thalamus, while a large signal loss and functional abnormalities have been found in these early visual areas (Barnes et al., 2001;Clavagnier et al., 2015;Hess et al., 2009;Wen et al., 2021). Thus, it is possible that the functional deficits in the early visual areas could be a result of abnormalities in feedback modulations.

In addition to monocular deficits, clinical and psychophysical studies also suggest abnormalities in binocular suppression (Hess et al., 2014;Zhou et al., 2018), consistent with electrophysiological evidence in animal models (Bi et al., 2011;Shooner et al., 2017). However, functional abnormalities in binocular interactions remain highly controversial in the neuroimaging studies of human amblyopia. In an EEG study, dichoptic masks showed no significant suppressive effect on visually evoked potentials for either the amblyopic or fellow eye (Baker et al., 2015). However, other neuroimaging studies found significant binocular suppression (Baker et al., 2015;Chadnova et al., 2017;Farivar et al., 2011;Lygo et al., 2021), but no significant difference between the two eyes (Chadnova et al., 2017). A recent fMRI study showed asymmetric suppressions between the amblyopic and fellow eyes, but the suppression was stronger from the amblyopic eye to the fellow eye than vice versa (Lygo et al., 2021), which is inconsistent with psychophysical (Zhou et al., 2018) and electrophysiological studies (Shooner et al., 2017).

**Fig. 1.**
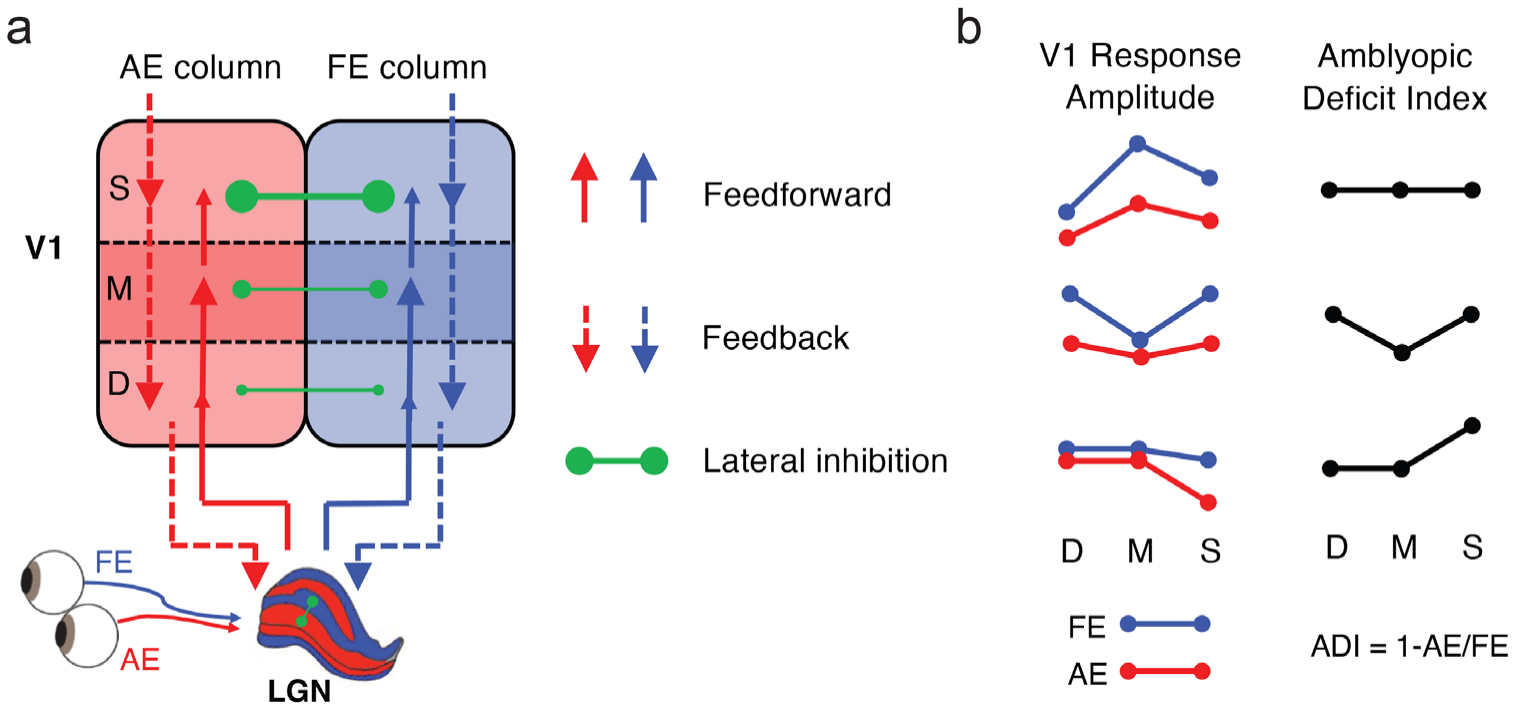
(a) Neural circuits in the geniculostriate pathway that may be affected by amblyopia. Solid and dashed arrows denote the feedforward and feedback connections, respectively. Green dots connected by a solid line indicate lateral inhibitions between adjacent ocular dominance columns. (b) Layer-specific response and amblyopic deficit in V1. Red and blue lines indicate AE and FE responses, respectively. The amblyopic deficit index (ADI) was defined as the proportion of response loss in AE compared with FE. S: superficial layers, M: middle layers, D: deep layers, AE: amblyopic eye. FE: fellow eye.

The primary visual cortex (V1) of primates consists of six layers of neurons with distinct roles in feedforward, feedback, and intracortical processing (Felleman & Van Essen, 1991). As illustrated by the laminar circuitry in V1 (Fig. 1a), input from the lateral geniculate nucleus (LGN) of the thalamus terminates mainly in the middle layer (4c) and with a smaller portion in the deep layer (6). The deep (5/6) and superficial layers (1/2/3) receive feedback projections from higher cortex. Horizontal or lateral inhibitions between ocular dominance columns (ODCs) are most prominent in the superficial layers (2/3) as demonstrated by electrophysiological recordings and intrinsic axonal connections (Cox et al., 2019;Gilbert & Wiesel, 1983;Sengpiel et al., 1995). Recent development of high-resolution fMRI at ultra-high magnetic fields (7 Tesla and above) allows to noninvasively measure cortical depth-dependent activity in humans (Fracasso et al., 2018;Huber et al., 2017;Kok et al., 2016;Liu et al., 2021), making it practically possible to study mesoscale deficits in cortical microcircuitry of feedforward, feedback, and binocular interactions. While a previous human fMRI study by Goodyears and colleagues examined amblyopic deficits at the level of cortical columns (Goodyear et al., 2002), mesoscale deficits across cortical depth remain unexplored.

In experiment 1 (Exp. 1), to investigate the cortical microcircuits underlying the amblyopic deficits, we used submillimeter resolution (0.8 mm isotropic) fMRI at 7 Tesla to measure layer-dependent responses in V1 of 10 human adults with unilateral amblyopia (seeSupplementary Table S1for clinical characteristics). As in previous electrophysiological studies in animal models (Kiorpes et al., 1998;Shooner et al., 2017), we defined an amblyopic deficit index (ADI) by the response difference between the amblyopic eye (AE) and the fellow eye (FE) divided by the FE response (Fig. 1b). In a linear system analysis for laminar fMRI (van Dijk et al., 2020), BOLD amplitude scales across cortical depth for stimuli with different signal strength from the thalamic input. Thus, if the AE activity loss arises from the thalamic input layers and propagates along the feedforward pathway, ADI would be comparable across cortical depths. Alternatively, if higher cortical deficits affect V1 processing through feedback modulations, ADI would be evident in the superficial and deep layers. Finally, in the binocular stimulus condition, stronger suppression from FE to AE than vice versa would produce a larger response deficit in the superficial layers of V1 by lateral inhibition.

In experiment 2 (Exp. 2), to further characterize the amblyopic abnormalities in binocular interactions and visual processing speed, we used an EEG frequency-tagging method to measure steady-state visually evoked potentials (SSVEPs) to stimuli presented to the two eyes at slightly different temporal frequencies (Norcia et al., 2015;Zhang et al., 2011). A total of 37 adults with unilateral amblyopia (seeSupplementary Table S1for clinical characteristics) and 15 healthy controls participated.

## 2 Methods

### 2.1 fMRI experiment (Exp. 1)

#### 2.1.1 Participants

A total of 10 adults diagnosed with unilateral amblyopia (9 anisometropia and 1 strabismus, 26.0 ± 7.90 years of age, 6 females) were enrolled in the fMRI experiment. Participants gave written informed consent approved by the institutional review board of the Institute of Biophysics, Chinese Academy of Sciences (2012-IRB-011). The research followed the tenets of the Declaration of Helsinki, and all participants gave written informed consent in accordance with procedures and protocols approved by the Human Subjects Review Committee of the Eye and ENT Hospital of Fudan University, Shanghai, China ([2023]No.202320). The best-corrected visual acuity (BCVA) of each subject was assessed by an experienced optometrist with the Snellen chart. Cover and alternative cover testing, duction and version testing, intraocular pressure testing, slit-lamp testing, indirect fundus examination after pupil dilation, and optometry were performed for each subject. The BCVAs of all amblyopic eyes were >0.1 logMAR and <1.0 logMAR, and the BCVA of all fellow eyes was ≤0.1 logMAR or better. Amblyopia patients were required to be free from a history of intraocular surgery, any eye diseases, and any systemic diseases known to affect visual function (e.g., migraine, congenital color deficiencies). None of amblyopia subjects underwent amblyopia treatment including patching treatment. All strabismic amblyopia patients had undergone strabismus surgery at least 1 year previously and had normal eye position and steady fixation. The clinical details of all patients are listed inSupplementary Table S1.

#### 2.1.2 Stimuli and procedures

**Fig. 2.**
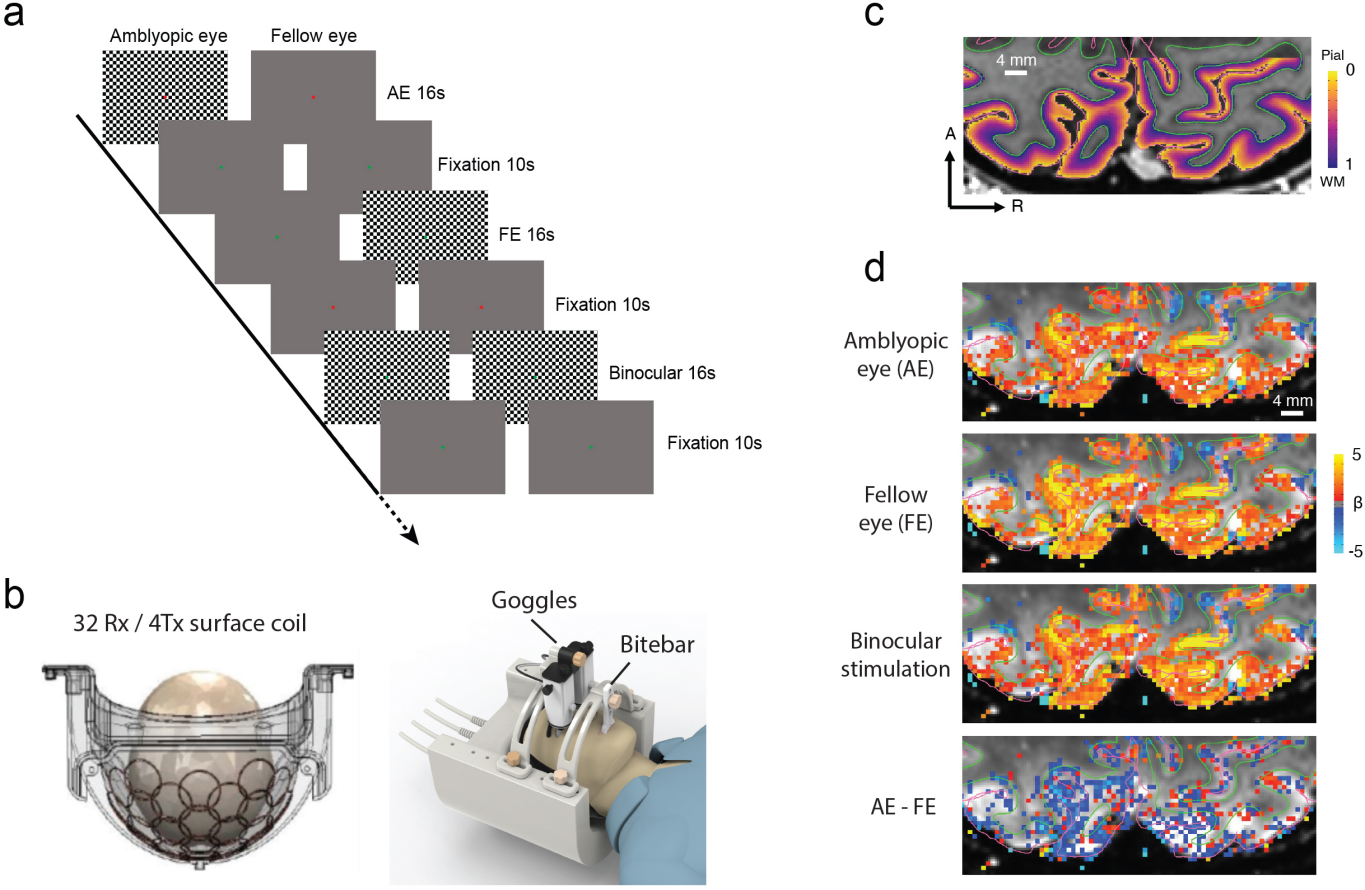
(a) Stimuli and procedure of the fMRI experiment. (b) The open-face surface coil, goggles, and bite bar. (c) Cortical depth map and segmentation in the occipital lobe of a representative subject (P10). Green and purple lines denote the white matter and gray matter surfaces. The underlay shows the structural image (T1-MP2RAGE). (d) Occipital activations to visual stimuli in the same subject. The underlay shows the mean functional image (GE-EPI). Maps were thresholded at p < 0.5, uncorrected.

In the 7T fMRI experiment (Exp. 1), a full contrast checkerboard (30-by-22.5 degrees of visual angle, 0.26 degrees checker size) counterphase flickering at 8 Hz was presented through a pair of MR-compatible goggles (NordicNeuroLab). The checkerboard flickers were presented to the amblyopic eye (AE), the fellow eye (FE), or both eyes (binocular) in separate blocks (18 s), interleaved with 12-s fixation blocks (Fig. 2a). Each run lasted 282 s, comprising 9 stimulus blocks (3 for each condition) and 10 fixation blocks. The order of stimulus conditions was pseudo randomized and counter balanced both within and across runs. During the experiment, subjects were instructed to keep fixating a central point. Both eyes wore glasses to correct for refractive errors.

#### 2.1.3 MRI data acquisition

MRI data were acquired with a 7T scanner (Siemens Magnetom) using a custom 32-channel receive 4-channel transmit open-face visual coil to acquire high-quality MRI data from the occipital and temporal lobes (Sengupta et al., 2016) (Fig. 1b), and a commercial 32-channel receive 1-channel transmit whole-brain head coil (NOVA Medical). A bite bar was used to reduce head motion. The open-face design enabled using both goggles and the bite bar. The gradient coil has a maximum amplitude of 70 mT/m, 200 μs minimum gradient rise time, and 200 T/m/s maximum slew rate.

High-resolution functional data were collected using a T2*-weighted 2D gradient-echo EPI sequence (0.8 mm isotropic voxels, TR = 2000 ms, TE = 23 ms, nominal flip angle = 78°, field of view 128 × 128 mm, 31 oblique-coronal slices without gaps covered the posterior part of the brain, receiver bandwidth = 1157 Hz/pix, phase partial Fourier = 6/8, GRAPPA acceleration factor = 3, phase encoding direction from A to P). It is worth to mention that the actual resolution of fMRI is lower than 0.8 mm due to T2* blur and phase partial Fourier. Two dummy scans were collected at the beginning of each run. Five EPI images with reversed phase encoding direction (P to A) were also acquired for EPI distortion correction before each run. T1-weighted anatomical volumes were acquired using a MP2RAGE sequence (0.7 mm isotropic voxels, FOV = 224 × 224 mm, 256 sagittal slices, TE = 3.05 ms, TR = 4000 ms, TI1 = 750 ms, flip angle = 4°, TI2 = 2500 ms, flip angle = 5°, bandwidth = 240 Hz/pix, phase partial Fourier = 7/8, GRAPPA = 3). For cortical segmentation, we collected whole-brain T1w MP2RAGE images with the same imaging parameters in a separate session using the NOVA coil.

#### 2.1.4 MRI data preprocessing

MRI data were preprocessed using AFNI (Cox, 1996) and the mripy package was developed in our laboratory (https://github.com/herrlich10/mripy). EPI volumes were corrected for slice timing, susceptibility distortion (blip-up/down method), head motion (6 parameters rigid body), and rescaled to percent signal change. To minimize the loss of spatial resolution, all spatial transformations were combined and applied in a single interpolation step (sinc method). Slow baseline drift and motion parameters were included as regressors of no interest in addition to the stimulus regressors in a general linear model (GLM). A canonical HRF (BLOCK4 in AFNI) was used in the GLM analysis.

#### 2.1.5 Laminar analysis

The outline of the data analysis pipeline is shown inSupplementary Figure S1. The whole-brain anatomical volume was segmented into white matter, gray matter, and CSF, based on which pial and white matter surfaces were reconstructed using FreeSurfer (v6.0) (Fischl, 2012) with -hires option. Results were visually inspected and manually edited.Figure 2cshows the segmentations of cortical gray and white matter in a representative subject.Figure 2dshows the visually evoked activation maps to AE, FE, and binocular stimuli, and AE-FE response for the same subject. The registration quality of all participants is shown inSupplementary Figure S2. To prevent the surface-to-volume projection from creating “holes” in high-resolution volumes, high-density meshes were generated by fourfold up-sampling from the default ones. The anatomical volume from the NOVA coil and the reconstructed surfaces were first aligned to the T1 image from the visual coil, and then to the mean EPI image. Relative cortical depths (0 at pial and 1 at white matter surface) of gray matter voxels were estimated using an equivolume method (Waehnert et al., 2014). Surface curvatures were considered in the depth estimation by taking the ratio of neighborhood areas for a pair of corresponding vertices on the pial and white matter surfaces. A set of intermediate surfaces were generated at various equivolume distances and the depth value for each voxel was estimated by linear interpolation between two nearest surfaces (mripy_align_anat.ipy from mripy). The superficial, middle, and deep layers were defined to each occupy one-third of the gray matter volume (Balaram et al., 2014;de Sousa et al., 2010;Liu et al., 2021). Cortical depth-dependent responses were averaged across all voxels in a layer compartment.

To reduce the influence from large veins on the BOLD laminar profiles, all voxels across cortical depth were excluded for each vertex contaminated by large veins. The bias-field corrected mean EPI intensity and averaged BOLD responses were mapped onto the cortical surface. Vertices with an EPI intensity lower than 70% of the averaged intensity or with a BOLD signal change over 10% were identified as large veins. These vertices were subsequently mapped back to the volumetric space to form a mask that encompassed all voxels across cortical depth. Voxels within this mask were excluded from subsequent analysis.

#### 2.1.6 ROI definition

Anatomical ROIs for early (V1, V2, V3) and intermediate (V4, LO, V3ab) visual areas were defined using a retinotopic atlas based on 7T fMRI data from the Human Connectome Project (Benson et al., 2018) within 5 degrees of eccentricity, corresponding to the central visual field of the stimulus. In the monocular condition, all vertices were included in the analyses. In the binocular condition, ocular biased vertices in V1 were defined as those with the most difference between AE and FE responses in the monocular condition (10% vertices from each side of the AE-FE beta distribution). In a supplementary analysis, we used a split-half-run approach to define the same ROIs for analyzing monocular and binocular responses (Supplementary Fig. S3). Data from the odd runs were used as an independent localizer to define the ocular biased vertices (using the same threshold as the above analysis) for analyzing monocular and binocular responses in the even runs. We then used the data from the even runs as an independent localizer to extract the responses from the odd runs. Results from the two split-half-run analyses were then averaged.

#### 2.1.7 Inverted encoding model

In the forward model, we hypothesized that each voxel’s fMRI response (Y) was driven by a linear combination of monocular channel responses (C) from the amblyopic eye and the fellow eye (Y = WC). The monocular channel mixing weights (W) for the two eyes could be solved using the fMRI responses during monocular stimulus conditions (for AE stimulation C_mono_= [1 0]^T^, for FE stimulation C_mono_= [0 1]^T^). The BOLD response for voxels preferring the non-stimulated eye could be weak and sometimes even negative in the presence of noise. However, the mixing weights should be non-negative. So we used non-negative least squares to solve W given Y_mono_and C_mono_in the monocular conditions. We then inverted the model to estimate the monocular channel responses in the binocular condition (C_bino_) using ordinary least squares, given the observed binocular response (Y_bino_) and channel mixing weights (W). Finally, the BSI for the two eyes was calculated as 1-C_bino_(equivalent to 1 – binocular/monocular channel responses) at each cortical depth.

### 2.2 EEG experiment (Exp. 2)

#### 2.2.1 Participants

A total of 37 adult patients diagnosed with unilateral amblyopia (21 anisometropic amblyopia, 4 strabismic amblyopia, 1 mixed amblyopia, and 1 deprived amblyopia after cataract surgery, 27.89 ± 5.94 years of age, 25 females) and 15 normal participants (25.20 ± 1.70 years of age, 10 females) were enrolled in the EEG experiment. The research followed the tenets of the Declaration of Helsinki, and all participants gave written informed consent in accordance with procedures and protocols approved by the Human Subjects Review Committee of the Eye and ENT Hospital of Fudan University, Shanghai, China ([2023]No.202320). Clinical examination and patient inclusion criteria were the same as in Exp. 1. Clinical details are listed inSupplementary Table S1. Eye dominance in normal subjects was determined by instructing the subjects to look at a distant letter with both eyes open through a hole between their hands, and then with their eyes alternatively closed. The eye that could still see the target was the dominant eye (hole-in-card method).

#### 2.2.2 Stimuli and procedures

In Exp. 2, visual stimuli were low- and high-pass filtered natural scene stimuli equalized in brightness and root mean square contrast (7 degrees of visual angle in diameter). Stimuli were on and off flickered at two temporal frequencies, 7.2 and 8 Hz, delivered one to each eye during monocular and binocular stimulus presentations. The association of tagging frequencies and eyes was balanced within each subject: 7.2 Hz for the left eye and 8 Hz for the right eye, and vice vera. Therefore, the EEG stimulation included a total of 2 SFs * 3 ocularities * 2 TFs = 12 stimulus conditions. Stimuli were dichoptically presented with a pair of base-out prism glasses and a cardboard divider splitting the left and right visual fields. A pair of circular frames with mosaic patterns outside the frames were used to assist fusion between the two eyes. Participants pressed a button to start a trial. They were instructed to keep fixation and avoid blinking as much as possible during the 6-s period of stimulus presentation. There were 20 stimulus blocks in the experiment, each had 12 stimulus conditions in a pseudo randomized order.

#### 2.2.3 EEG data collection

EEG data were collected with a 64-channel system (BrainVision actiCHamp), digitized at 1000 Hz. The ground electrode was located in front of AFz, and the reference electrode between Fz and Cz. Electrode impedance was maintained below 8 kΩ throughout the experiment. A DELL S2721DGF monitor was used for stimulus presentation, setup at 144 Hz refresh rate and 2560*1440 resolution. The distance between the monitor and the eyes of participants was 85 cm.

#### 2.2.4 EEG data analysis

EEG data were preprocessed using EEGLAB in Matlab. For SSVEP amplitude analysis, a bandpass filter between 1 and 30 Hz was applied on the raw EEG time series. To avoid the transient response from stimulus onset, we used the last 5 s of data in each trial for further analysis. Since SSVEPs are less susceptible to low frequency artifacts such as blinks and eye movement, we included all trials in the following analysis (Vialatte et al., 2010). Mean time series were generated by averaging across all trials in each condition. A spatial Laplacian filter was used to increase the signal-to-noise ratio, with O1, Oz, O2 and PO3, POz, PO4 as the central electrodes, and P5, P3, P1, Pz, P2, P4, P6 and PO7, PO8 as the surround electrodes. The differential signal between the means of central and surround electrodes was fast Fourier transformed to obtain the amplitude spectrum. Amplitudes of the first- and the second-order harmonics were summed together. The amplitudes of SSVEPs to the 7.2 and 8 Hz stimuli were averaged in each condition. For the phase analysis, a bandpass filter with a 6–9 Hz frequency range was applied to the raw time series. EEG data from 1251 to 5000 ms (integral multiples of periods at both stimulus frequencies) after stimulus onset were used. A least squares filtering method was used to calculate the phase of mean time series at the stimulus frequency.

#### 2.2.5 Fixation stability

The fixation stability of the amblyopic eye was assessed using an EyeLink 1000 eye tracker (SR Research). Participants sat a viewing distance of 50 cm, with their head position stabilized with a chin rest. A red fixation dot (0.34 degrees in diameters) was presented on a black background. Participants were instructed to maintain fixation with the fellow eye patched. Each run lasted 42 s. A total of three runs of data were collected.

### 2.3 Statistics

Statistical tests were performed using Pingouin (v0.5.4) and JASP (v0.14.1). If not otherwise specified, the comparison of mean responses between conditions, layers, or groups was tested for significance using ANOVA followed by two-tailed t-test. If multiple measurements were taken from the same group under different conditions or from different ROIs, repeated measures ANOVA and paired t-test were used. The follow-up tests were corrected for multiple comparisons using the Holm method, unless there were only three levels followed by a significant one-way ANOVA, or two levels followed by a significant 2 x 2 interaction, in which cases a correction is not necessary (Cohen, 2008;Levin et al., 1994). Partial eta squared and Cohen’s d were reported as the effect size for ANOVA and t-test, respectively. When comparing BSI within and between groups, non-parametric methods (Wilcoxon signed-rank test for paired tests and Wilcoxon rank-sum test for independent samples test) were used due to the large difference in variance. Rank-biserial correlation (RBC) was reported as effect size. Error bars in the bar plots indicate 95% confidence interval, which was generated using a bootstrap method (seaborn v0.12.2).

We adopted a multivariate approach in detecting data outliers. For each set of comparisons, the measurements from the same subject were considered as a single point in a multidimensional space, and the points whose likelihood was less than 1% (assuming an underlying multivariate normal distribution) were flagged as outliers (encircled by red outlines inFigs. 4c, dand5) and excluded from subsequent statistical tests. The likelihood was obtained by evaluating the Mahalanobis distance (squared) of the point against a chi-squared distribution with degrees of freedom equal to the number of dimensions. The phase differences shown inFigure 6were tested using the Circular Statistics Toolbox (v1.21) in Matlab (Berens, 2009). “circ_mtest” was used to test the within-subject phase delay between stimulus conditions. “circ_wwtest” was used to test the phase delay between AE/FE and NE.

## 3 Results

### 3.1 Amblyopic response deficits in different cortical depths of V1 and in higher visual cortices

**Fig. 3.**
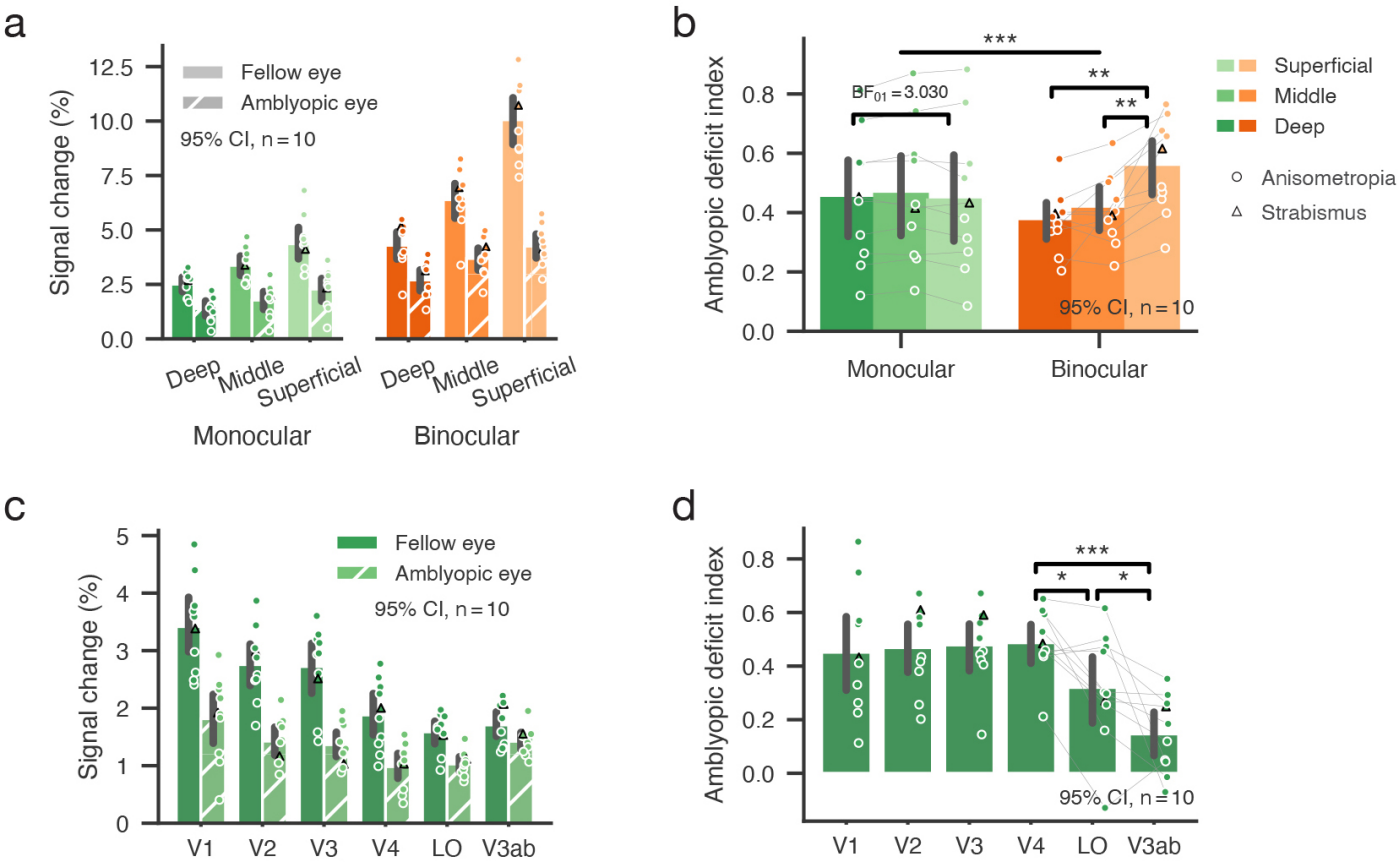
(a) Cortical depth-dependent fMRI responses in V1 (group-averaged across 10 participants). (b) Amblyopic deficit indices in the monocular and binocular stimulus conditions across cortical depths in V1. (c) BOLD responses to monocular stimuli in V1 and higher cortical regions. (d) Monocular ADIs in the visual cortical areas. In all panels, each dot represents datum from one participant (circle for anisometropia, triangle for strabismus). *, **, and *** denote p < 0.05, 0.01, and 0.001, respectively. Error bars represent 95% confidence intervals across participants.

Group-averaged BOLD responses in V1 showed a strong bias toward the superficial depth in both monocular and binocular stimulus conditions (Fig. 3a), consistent with influence from intracortical draining veins and large pial veins (Havlicek & Uludağ, 2020;Huber et al., 2019). Using the amplitude-normalized amblyopic deficit index (ADI) was able to alleviate such limitation, and our results indeed showed distinct laminar profiles in the monocular and binocular stimulation conditions (Fig. 3b), supported by a highly significant interaction between ocularity (monocular/binocular) and layers (superficial/middle/deep) in a two-way repeated measures (rm) ANOVA (F(2,18) = 19.068, p < 0.001, η^2^_p_= 0.680). Monocular ADIs were comparable across cortical depths (F(2,18) = 1.519, p = 0.246, BF_01_= 1.885), while the binocular ADI was strongest at the superficial depth (F(2,18) = 14.224, p < 0.001, η^2^_p_= 0.612; S vs. M: t(9) = 4.250, p = 0.002, Cohen’s d = 1.002; S vs. D: t(9) = 4.275, p = 0.002, Cohen’s d = 1.361). To ensure high signal-to-noise ratio in the depth-dependent analysis, monocular ROIs on the cortical surface were defined as a large ROI within 5 degrees of eccentricity (corresponding to the central visual field of the stimulus) based on a retinotopic atlas of the Human Connectome Project (Benson et al., 2014,^2018^). According to the previous study (Benson et al., 2018), retinotopic atlas of early visual areas (V1–V3) aligned very well across participants on the standard surface using surface-based registration by anatomical landmarks. Binocular ROIs were defined as vertices showing strong ocular bias in the monocular stimulus conditions. To ensure unbiased sampling across cortical depth, all voxels spanning the cortical depth were included for each vertex in the voxel space (see Methods 2.1.5 for details). In a further analysis, we also defined the same ROIs for the monocular and binocular conditions using a split-half approach. The difference in the laminar profiles of monocular and binocular ADIs was still highly significant (p < 0.001), with the binocular ADI more biased to the superficial depth (Supplementary Fig. S3). Although there was only one strabismus patient, the laminar pattern was highly similar to the anisometropic participants.

Monocular response deficit was also found in higher cortical regions beyond V1 (Fig. 3c). Monocular ADIs were comparable in the early visual areas from V1 to V3 (Fig. 3d; F(2,18) = 0.131, p = 0.878, BF_01_= 4.333), but there was a significant ventral-to-dorsal gradient of alleviated deficits in the middle visual areas V4, LO, and V3ab (F(2,18) = 14.710, p < 0.001, η^2^_p_= 0.620; V4 vs. LO: t(9) = 3.065, p = 0.013, Cohen’s d = 0.969; V4 vs. V3ab: t(9) = 5.842, p < 0.001, Cohen’s d = 2.559; LO vs. V3ab: t(9) = 2.349, p = 0.043, Cohen’s d = 0.968). These findings support that the amblyopic deficit in monocular processing arises from the thalamic input layers of V1 and carries over to the ventral stream in feedforward processing (Fig. 1). In the binocular viewing condition, stronger suppression from the fellow eye leads to an additional response loss in the superficial layer of V1 by lateral inhibition.

Due to the partial volume effect of fMRI, voxels biased to one eye were influenced by signals from the opposing eye, even at the submillimeter resolution. This can lead to an underestimation of binocular ADIs inFigure 3b(compared with the EEG results inFig. 5c). The partial volume effect poses a challenge to measure the effect of binocular suppression, since the binocular response could be even stronger than the monocular response in ocular biased voxels. To alleviate the partial volume effect, we used an inverted encoding model (IEM) method to unmix the monocular channel responses in the binocular stimulus condition (seeMethods 2.1.7for details) (Brouwer & Heeger, 2009). The IEM results are shown inFigure 4. For the fellow eye, the binocular suppression index (BSI = 1 – binocular/monocular channel response) was close to zero and was significantly smaller than that of the AE (main effect of eye F(1,9) = 33.000, p < 0.001, η^2^_p_= 0.786). The BSI for the FE was lowest in the superficial layers (main effect of layer F(2,18) = 14.854, p < 0.001, η^2^_p_= 0.623; S vs. M: t(9) = -3.140, p = 0.012, Cohen’s d = 0.139; S vs. D: t(9) = -4.705, p = 0.001, Cohen’s d = 0.340), whereas the BSI for the AE increased toward the superficial cortical depth (F(2,18) = 8.946, p = 0.002, η^2^_p_= 0.498; S vs. M: t(9) = 2.570, p = 0.030, Cohen’s d = 0.369; S vs. D: t(9) = 3.537, p = 0.006, Cohen’s d = 0.610). The increased imbalance in binocular suppression in the superficial layers parallels the laminar profile of amblyopic deficit index (ADI) inFigure 3b.

**Fig. 4.**
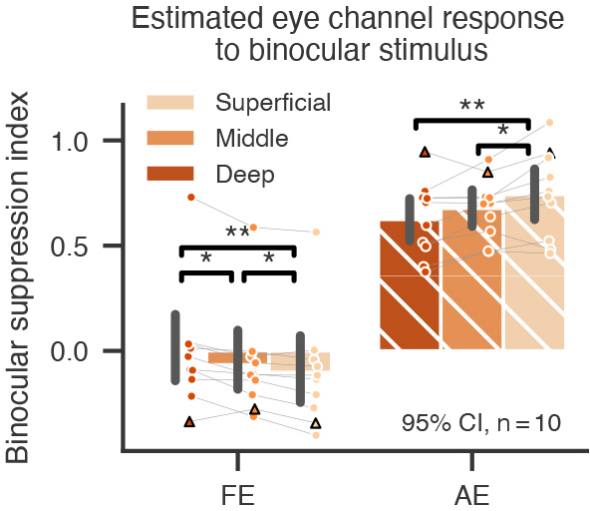
Binocular suppression index (BSI) for the fellow eye and the amblyopic eye as a function of cortical depth. BSIs were calculated by an IEM method (seeMethods 2.1.7for details). Each dot represents datum from one participant (circle for anisometropia, triangle for strabismus). * and ** denote p < 0.05 and 0.01, respectively. Error bars represent 95% confidence intervals across participants.

**Fig. 5.**
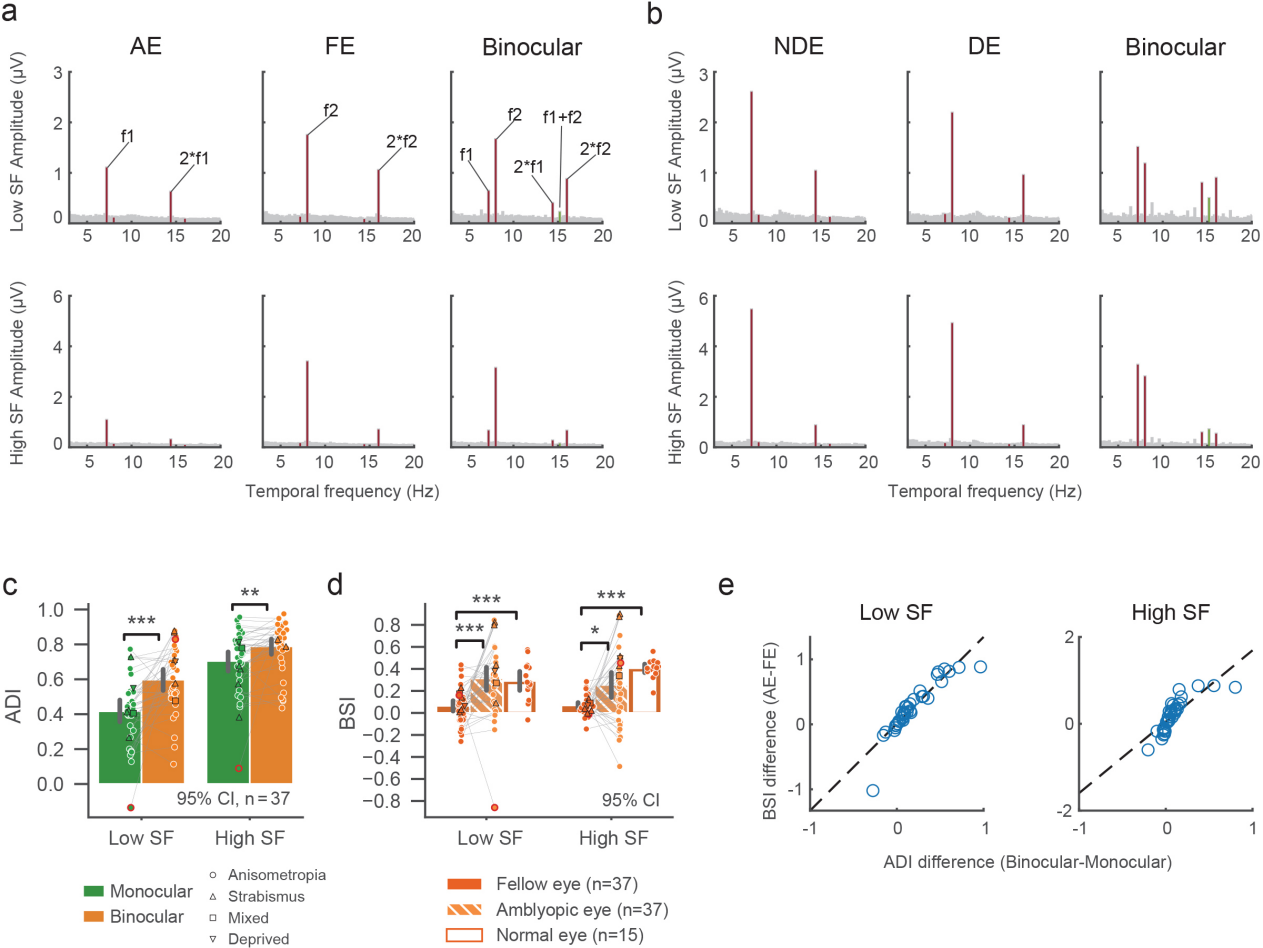
(a) Amplitude spectrums of SSVEPs in monocular and binocular conditions averaged across all amblyopic subjects. The stimulus frequencies in AE and FE were f1 = 7.2 Hz and f2 = 8 Hz, respectively.Supplementary Figure S4shows results from the opposite setup (f2 in AE and f1 in FE). Green lines represent the intermodulation frequency at f1 + f2. Upper and lower rows show the results for low and high spatial frequency (SF) stimuli, respectively. (b) SSVEP amplitude spectrum in normal controls (f1 in non-dominant eye (NDE) and f2 in dominant eye (DE); SeeSupplementary Figure S4for f2 in NDE and f1 in DE). (c) Amblyopic deficit index (ADI) for both monocular (green) and binocular (orange) stimulus conditions. Different symbols represent individuals with different types of amblyopia: anisometropia (circle), strabismus (triangle up), mixed (square), deprived (triangle down). Symbols with red outline are detected as outlier based on within-group multivariate Mahalanobis distance (see Methods) and excluded from reported statistics. Error bars represent 95% confidence intervals. *p < 0.05, **p < 0.01, ***p < 0.001. (d) Binocular suppression index (BSI) for the FE (solid), AE (dashed), and normal eye (NE, unfilled), otherwise same convention as in (c). (e) Correlations between the difference in ADI (binocular-monocular) and in BSI (AE-FE). Open circles represent data from individual subjects.

Although the IEM method can help unmix monocular channel responses under the binocular stimulus condition, it relies on the assumption that channel responses combine linearly based on fixed monocular channel weights—an assumption that may not hold entirely. Thus, in order to further investigate the effect of binocular suppression and its relationship with the amblyopic deficits, we used an SSVEP frequency-tagging method in Exp. 2 to measure the two eyes’ responses to naturalistic stimuli presented at slightly different temporal frequencies. Visual stimuli were low- and high-pass filtered to selectively evoke neural activity biased to the magnocellular (M) and parvocellular (P) channels, respectively. The cutoff frequency (2 c.p.d.) was determined based on the contrast sensitivity functions of the parallel pathways in primates (Merigan, Byrne, et al., 1991;Merigan, Katz, et al., 1991;Merigan & Maunsell, 1993). It is known that the amblyopic eyes have reduced fixation stability (Chung et al., 2015;Kelly et al., 2019). A salient fixation point and a fusion frame oval on mosaic background were presented binocularly to help maintain fixation stability (Gonzalez et al., 2012). We also monitored eye movements in a pilot test to ensure that patients can maintain fixation (1.24 (mean) ± 1.01 (std) deg^2^of bivariate contour ellipse area, compared with 7 degrees of stimulus size).

### 3.2 Reduced suppression from AE correlates with binocular response deficits

As shown inFigure 5a and b, the amplitude spectrum of SSVEPs in both amblyopic and control groups showed high signal-to-noise ratio at the stimulus frequencies. Binocular ADIs were significantly larger than monocular ADIs (Fig. 5c), demonstrated by a significant main effect of ocularity (F(1,35) = 18.898, p < 0.001, η^2^_p_= 0.351) in a two-way rm ANOVA. This effect was significant in both low (t(35) = -4.336, p < 0.001, Cohen’s d = -0.895) and high (t(35) = -3.027, p = 0.005, Cohen’s d = -0.431) spatial frequency (SF) conditions, but more robust in low than in high SF (F(1,35) = 9.491, p = 0.004, η^2^_p_= 0.213). We also computed a binocular suppression index (BSI), defined as the amplitude difference between monocular and binocular conditions divided by the monocular amplitude (1-binocular/monocular), to indicate the degree of suppression by the opposing eye (Fig. 5d). For patients with amblyopia, BSI was significantly larger for AE than for FE (F(1,35) = 22.213, p < 0.001, η^2^_p_= 0.388), in both low (Wilcoxon signed-rank test W = 44, p < 0.001, rank-biserial correlation (RBC) = -0.868) and high (W = 162, p = 0.019, RBC = -0.514) SF conditions. This finding is consistent with the BSI results of fMRI inFigure 4. In healthy controls, BSIs showed no significant difference between the dominant (DE) and non-dominant (NDE) eyes, thus were averaged as the normal eye (NE) group. In the low SF condition, BSI for NE was significantly larger than that for FE (Wilcoxon rank-sum test U = 84, p < 0.001, RBC = -0.689) but close to AE (U = 310, p = 0.414, RBC = 0.148). Similarly in the high SF condition, BSI for NE was significantly larger than that for FE (U = 3, p < 0.001, RBC = -0.989) but not than that for AE (U = 170, p = 0.079 after Holm correction, RBC = -0.370).

These findings provide strong evidence for asymmetric binocular suppressions between AE and FE, that is, stronger suppression from FE to AE than vice versa. This was mainly due to reduced suppression by AE compared with binocular suppressions in the control group. To further investigate whether the asymmetric binocular suppression is related to the additional deficits in the binocular condition, we calculated Pearson’s correlation between the difference in ADI (binocular–monocular) and in BSI (AE-FE). Results showed a highly significant correlation in both low (r = 0.895, p < 0.001) and high (r = 0.743, p < 0.001) SF conditions, supporting that the additional ADI in the binocular condition is a result of asymmetric binocular suppression between AE and FE.

### 3.3 Reduced intermodulation amplitude indicates weakened binocular integration

**Fig. 6.**
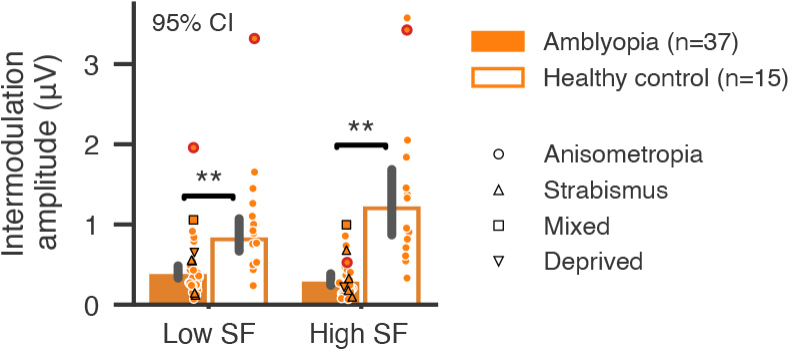
SSVEP amplitudes at the intermodulation frequency (f1 + f2). The symbols follow the same conventions as inFig. 5candd. Error bars represent 95% confidence intervals. ** denotes*p*< 0.01.

To further investigate functional abnormalities in binocular integration, we examined the difference in the amplitude of intermodulation (IM) frequency (f1 + f2) between amblyopes and healthy controls (Fig. 6). IM amplitudes were significantly reduced in the amblyopic group (F(1,48) = 44.601, p < 0.001, η^2^_p_= 0.482), and in both high and low SF conditions (high SF: t(13.784) = -4.159, p = 0.002, Cohen’s d = -1.973; low SF: t(16.384) = -3.946, p = 0.002, Cohen’s d = -1.572). Moreover, there was a significant SF × group interaction (F(1,48) = 12.901, p < 0.001, η^2^_p_= 0.212), suggesting larger deficit in binocular integration in the high SF condition.

### 3.4 Delayed monocular and binocular processing in the early visual cortex

To investigate the amblyopic deficits in visual processing speed, we analyzed the phase of SSVEPs in AE, FE, and NE conditions using a least-squares filter method (Tang & Norcia, 1995). The group averaged SSVEP time courses inFigure 7ashow clear phase delays between AE and FE, and between FE and NE. In addition, neural activity to high SF stimuli was also clearly delayed compared with the low SF activity.

**Fig. 7.**
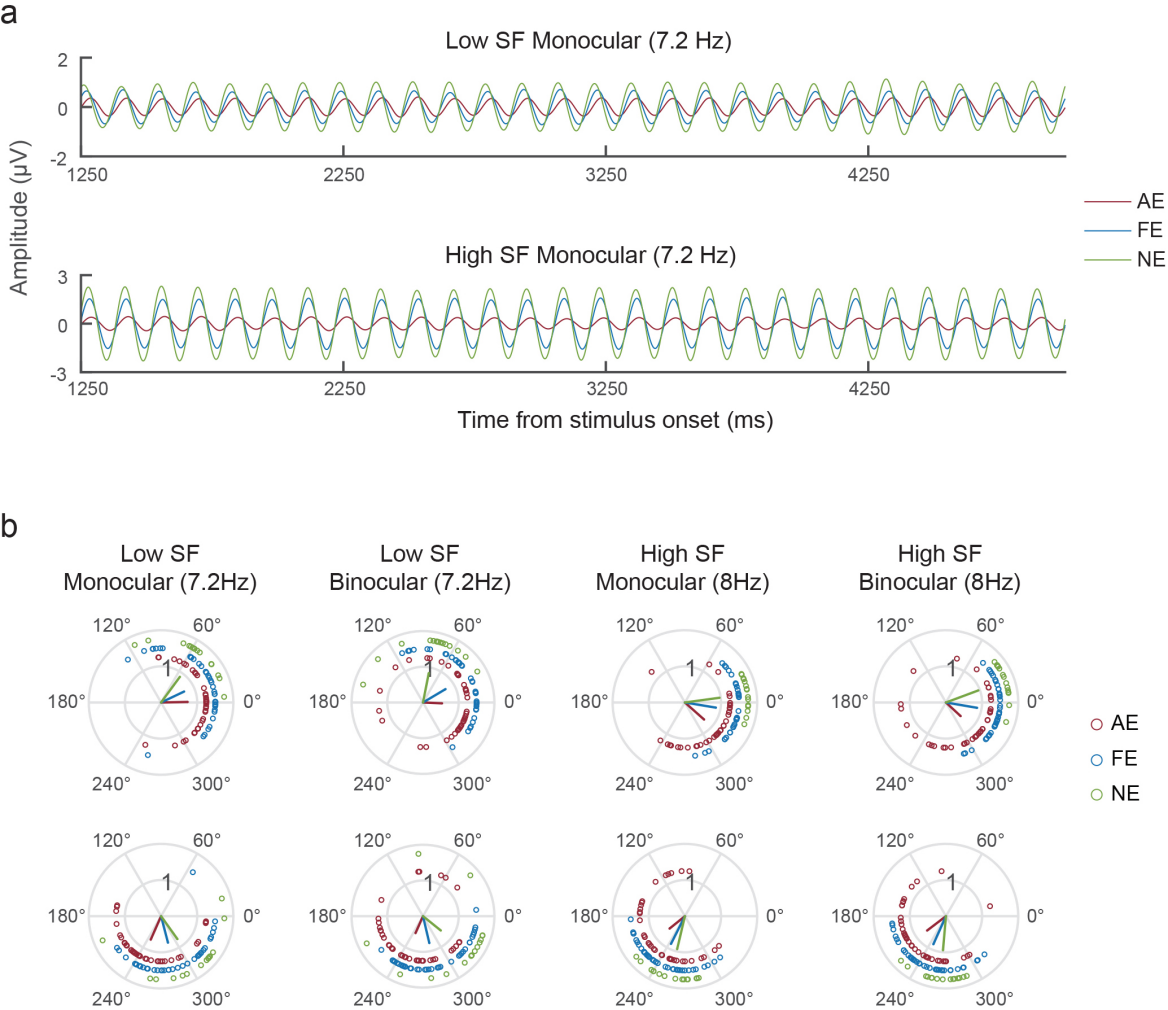
(a) Group averaged SSVEPs. The monocular stimulus conditions at 7.2 Hz are shown here as examples (from 1250 to 5000 ms after stimulus onset). (b) Polar plots of SSVEP phases. Red, blue, and green lines denote group averaged phases for AE, FE, and NE responses. Circles represent individual data. DE and NDE phases were averaged for the NE group.

The polar plot shown inFigure 7billustrates the phase of SSVEPs in all stimulus conditions. Using circular statistics (Berens, 2009), we found a significant phase delay between AE and FE (circ_mtest, p < 0.001, mean delay = 12.81 ms), and between AE and NE (Watson–Williams test, p < 0.001, mean delay = 21.69 ms). FE responses were also significantly delayed relative to NE (Watson–Williams test, p = 0.003, mean delay = 8.88 ms). Neural responses to binocular stimuli were also significantly faster than those to the monocular stimulus conditions in normal controls (circ_mtest, p < 0.001, mean delay = -6.24 ms), but not in amblyopic subjects (AE: p = 0.237, mean delay = -0.98 ms; FE: p = 0.721, mean delay = -0.089 ms). This aligns with our finding of impaired binocular integration indexed by reduced intermodulation amplitude. Finally, high SF responses were significantly delayed compared with the low SF conditions (circ_mtest, FE: p < 0.001, mean delay = 14.27 ms; NE: p < 0.001, mean delay = 20.87 ms; AE: p = 0.022, mean delay = 9.27 ms), consistent with slower processing speed of the parvocellular pathway.

## 4 Discussion

Using submillimeter 7T fMRI and EEG frequency-tagging methods, we investigated the neural deficits in mesoscale neural circuitry of monocular processing and binocular interactions in human adults with unilateral amblyopia. Monocular response deficits in V1 were consistent across cortical depths and comparable with those observed in extrastriate visual areas (V2–V4). This suggests that the amblyopic deficit originates in cortical layers receiving thalamic input and is subsequently propagated to downstream visual areas through feedforward processing. In the binocular stimulus condition, a stronger response deficit and binocular suppression was observed in the superficial layers of V1, consistent with lateral inhibition and suppression by the fellow eye. EEG results further revealed reduced suppression from the amblyopic eye, weakened binocular integration, and delayed monocular and binocular processing. These findings, with mesoscopic and millisecond resolution, elucidate the precise neural deficits underlying monocular processing and binocular interactions in human amblyopia. Although most of our participants were anisometropic amblyopia, the pattern of results for other types of amblyopia was highly similar.

### 4.1 Amblyopic deficits in monocular processing

The locus of amblyopic deficits has been a long-standing question in the literature. In addition to low-level monocular deficits in visual acuity and contrast detection, psychophysical studies also suggest abnormalities in higher visual functions (Levi, 2006,^2020^;Sharma et al., 2000), such as second-order detection (Wong et al., 2001), contour and motion integration (Hess et al., 1997;Simmers et al., 2003), shape discrimination (Hess et al., 1999), and attention (Popple & Levi, 2008;Verghese et al., 2019). Neural deficits of amblyopia at multiple levels of the visual hierarchy have also been demonstrated using electrophysiological recordings in animal models (Kiorpes, 2006,^2019^), and BOLD fMRI in humans (Anderson & Swettenham, 2006;Lerner et al., 2003;Muckli et al., 2006). The current consensus is that the neural deficit of amblyopia originates in V1, potentially causing additional functional impairments in downstream visual areas (Anderson & Swettenham, 2006;Kiorpes, 2019;Levi, 2020). However, it remains unclear whether disruptions in hierarchical visual processing in amblyopia involve feedforward processes, feedback processes, or both. Using simple checkerboard stimuli, our 7T fMRI results support that monocular deficits of amblyopia in the visual cortex are detectable from the outset at the thalamic input layer of V1 and cascade to downstream areas along the feedforward pathway. This is also consistent with the functional and structural abnormalities found in the LGN of the thalamus in human amblyopia (Barnes et al., 2010;Hess et al., 2010;Wen et al., 2021). However, the current findings using simple checkerboard stimuli do not preclude the possibility of abnormal feedback processing with more complex visual stimuli.

### 4.2 The role of suppression in amblyopic deficits and abnormalities in binocular integration

In addition to the neural deficits in monocular processing, binocular suppression may also play important roles in the visual deficits of amblyopia as suggested by clinical evidence (DeSantis, 2014;Von Noorden, 1996) and psychophysical studies (Baker et al., 2008;Holopigian et al., 1988;Li et al., 2011;Zhou et al., 2013). For example, using dichoptic motion detection, Li and colleagues found a negative correlation between the strength of binocular suppression and the depth of amblyopia (Li et al., 2011). More recent psychophysical evidence showed reduced suppression from the amblyopic eye relative to normal controls (Zhou et al., 2018). In animal models with non-human primate, there was a striking loss of binocular neurons in V1 (Kiorpes, 2019;Kiorpes et al., 1998;Movshon et al., 1987). Stronger than normal binocular suppression of neuronal activity has also been observed in the early visual cortex of strabismic monkeys (Bi et al., 2011), in which the suppression from FE to AE was stronger than that from AE to FE (Shooner et al., 2017). However, in the neuroimaging studies of human amblyopia (Baker et al., 2015;Chadnova et al., 2017;Farivar et al., 2011;Lygo et al., 2021), the suppression effect was either absent or not consistent with the behavioral and electrophysiological studies. Such inconsistency might be due to the difficulty to study binocular interactions at a conventional fMRI resolution, or a small number of participants.

Using submillimeter 7T fMRI and EEG frequency-tagging techniques in a relatively large group of participants, we were able to study binocular interactions between monocular channels and provide robust evidence for abnormal binocular interactions in human amblyopia. In contrast to a flat laminar profile in monocular response deficit, the amblyopic deficit in binocular responses, though underestimated due to partial volume effect, was strongest in the superficial depth of V1 (Fig. 3b), suggesting an additional signal loss due to suppression from the fellow eye by lateral inhibition. The IEM fMRI results (Fig. 4) and the SSVEP results from the EEG experiment (Fig. 5c, d) further demonstrate that the additional response deficit in the binocular condition was a result of imbalanced binocular suppression in the early visual cortex. This was mainly due to reduced suppression by the amblyopic eye relative to normal controls, consistent with recent psychophysical evidence (Gong et al., 2020;Zhou et al., 2018). In addition, we also found a significant reduction in the intermodulation amplitude. The IM frequency component could only arise from nonlinear combinations of the two eyes’ signals (Baitch & Levi, 1988;Regan & Regan, 1988). Recent studies suggest that the IM component may reflect interocular suppression in binocular vision (Du et al., 2023;Katyal et al., 2018). Therefore, a reduction in the IM amplitude suggests weakened binocular interactions in amblyopic subjects. This finding was also consistent with our finding of reduced suppression from the amblyopic eye (Fig. 4), the loss of binocular neurons found in the monkey studies (Kiorpes, 2019), and a recent study showing a correlation between IM amplitude and the interocular difference in contrast sensitivity (Gu et al., 2020). Based on neuroanatomical and electrophyiological evidence (Cox et al., 2019;Dougherty et al., 2019;Hubel & Wiesel, 1972), binocular neurons mainly exist in the superficial layers of V1, combining the two eyes’ signals through nonlinear normalizations (Mitchell et al., 2023;S.-H. Zhang et al., 2024). Our findings thus filled the gap between the psychophysical studies in humans and electrophysiological studies in the animal models about the abnormalities of amblyopia in binocular interactions.

### 4.3 Amblyopic deficits in visual processing speed

Delayed visual processing to stimulus presented to the amblyopic eye relative to the fellow eye has been reported in two previous studies (Chadnova et al., 2017;Lygo et al., 2021). However, the difference from normal controls remains unclear. In the current study (Fig. 7), significant phase delays of SSVEPs were observed not only between AE and NE, but also between FE and NE, in both monocular and binocular stimulus conditions. These findings demonstrate slower visual processing speed for both amblyopic and fellow eyes relative to normal controls. The phase delay was unlikely due to reduced neural activity, since the P-biased high SF responses exhibited significant phase delay, but much larger response amplitude than the M-biased low SF responses. For the control group, binocular stimuli also showed faster response timing than monocular stimuli, suggesting a benefit in visual processing speed with binocular vision. However, the binocular advantage in visual processing speed was not observed in AE or FE responses in amblyopic subjects. This finding provides additional support for amblyopic abnormalities in binocular integration, consistent with the reduction in intermodulation amplitude (Fig. 6). Therefore, our data present robust electrophysiological evidence for delayed visual processing in both the amblyopic and fellow eyes compared with normal controls. Weakened binocular integration, most likely in the superficial layers of V1, further reduced visual processing speed in binocular vision.

## 5 Conclusion

**Fig. 8.**
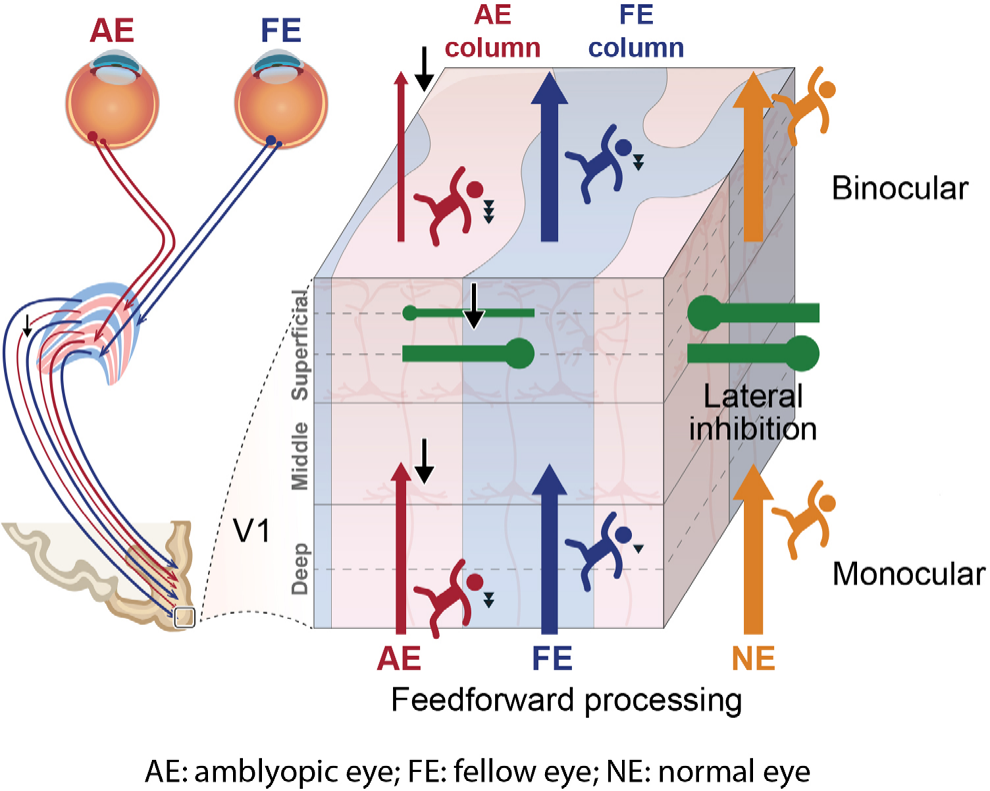
Attenuated and delayed neural activity in the cortical microcircuitry of human amblyopia. Black arrows and triangles indicate the amblyopic deficits in neural signal strength and visual processing speed, respectively.

Using a combination of submillimeter 7T fMRI and EEG frequency-tagging methods, we found robust evidence for functional abnormalities in the cortical microcircuitry of monocular processing and binocular interactions in human amblyopia (Fig. 8). Monocular processing showed attenuated and delayed neural activity in V1 layers receiving thalamic input, followed by imbalanced binocular suppression and weakened binocular integration in the superficial layers, which further reduced visual signal strength and processing speed. These findings demonstrate how abnormal visual experience during the critical period influences the development of cortical microcircuitry in humans. The precise neural deficits at mesoscopic and millisecond resolution may also help to develop more targeted and effective treatments, such as dichoptic anti-suppression therapy to improve visual signal strength and processing speed in amblyopic patients.

## Supplementary Material

Supplementary Material

## Data Availability

Data and code to reproduce the main findings of this study can be requested by contacting the corresponding author P.Z. The mripy package for high-resolution fMRI data processing can be downloaded from github (https://github.com/herrlich10/mripy).
