# Supplementary material for: Attenuated and delayed neural activity in cortical microcircuitry of monocular processing and binocular interactions in human amblyopia
Source: Imaging Neurosci (Camb). 2025 Apr 30;3:imag_a_00561. doi: 10.1162/imag_a_00561 (PMC12319873; doi:10.1162/imag_a_00561)
Supplement: Supplementary Material [file imag_a_00561-supp.pdf]

Table S1. Clinical details of amblyopic subjects

Subject ID	Age	Gender	Handedness	BCVA (logMAR)	Type of amblyopia	Eye alignment
7T P01	37	F	right	1.0	anisometropia	orthotropia
7T P02	28	M	right	1.0	anisometropia	orthotropia
7T P03	19	M	right	1.0	anisometropia	orthotropia
7T P04	20	F	right	0.7	anisometropia	orthotropia
7T P05	23	M	right	0.5	strabismic	Orthotropia (post surgery)
7T P06	16	M	right	1.1	anisometropia	orthotropia
7T P07	33	F	right	0.5	anisometropia	orthotropia
7T P08	17	F	right	0.4	anisometropia	orthotropia
7T P09	33	F	right	0.3	anisometropia	orthotropia
7T P10	34	F	right	0.8	anisometropia	orthotropia
EEG P01	29	F	Left	0.4	anisometropia	orthotropia
EEG P02	25	M	Left	0.3	anisometropia	orthotropia
EEG P03	26	F	Right	0.6	anisometropia	orthotropia
EEG P04	34	F	Right	0.7	anisometropia	orthotropia
EEG P05	23	M	Left	0.7	anisometropia	orthotropia
EEG P06	21	F	Left	0.3	anisometropia	orthotropia
EEG P07	25	F	Left	0.4	anisometropia	orthotropia
EEG P08	30	F	Right	0.4	anisometropia	orthotropia
EEG P09	30	F	Right	0.3	strabismus	orthotropia (post surgery)
EEG P10	25	F	Left	0.5	anisometropia	orthotropia
EEG P11	28	M	Left	0.3	anisometropia	orthotropia
EEG P12	30	F	Right	0.4	anisometropia	orthotropia
EEG P13	30	F	Right	0.5	strabismus	orthotropia (optical correction)
EEG P14	26	M	Right	0.8	anisometropia	orthotropia
EEG P15	18	F	Right	0.2	anisometropia	orthotropia
EEG P16	29	M	Left	0.1	anisometropia	orthotropia
EEG P17	26	F	Left	0.5	anisometropia	orthotropia
EEG P18	34	F	Left	0.3	anisometropia	orthotropia
EEG P19	25	F	Left	0.5	anisometropia	orthotropia
EEG P20	37	F	Right	0.7	anisometropia	orthotropia

EEG P21	29	F	Left	0.5	anisometropia	orthotropia
EEG P22	21	M	Left	0.7	anisometropia	orthotropia
EEG P23	18	F	Left	0.7	mixed	orthotropia (post surgery)
EEG P24	23	M	Left	0.7	deprived	orthotropia (post surgery)
EEG P25	34	F	Left	0.7	anisometropia	orthotropia
EEG P26	21	F	Left	0.4	anisometropia	orthotropia
EEG P27	37	M	Right	0.6	anisometropia	orthotropia
EEG P28	27	F	Right	0.5	anisometropia	orthotropia
EEG P29	24	F	Right	0.3	anisometropia	orthotropia
EEG P30	23	M	Right	0.8	anisometropia	orthotropia
EEG P31	22	M	Right	0.5	anisometropia	orthotropia
EEG P32	22	F	Left	0.4	anisometropia	orthotropia
EEG P33	35	M	Left	0.4	strabismus	orthotropia (optical correction)
EEG P34	30	F	Left	0.7	anisometropia	orthotropia
EEG P35	33	F	Right	0.5	anisometropia	orthotropia
EEG P36	39	M	Right	0.2	strabismus	orthotropia (optical correction)
EEG P37	43	F	Left	0.5	anisometropia	orthotropia

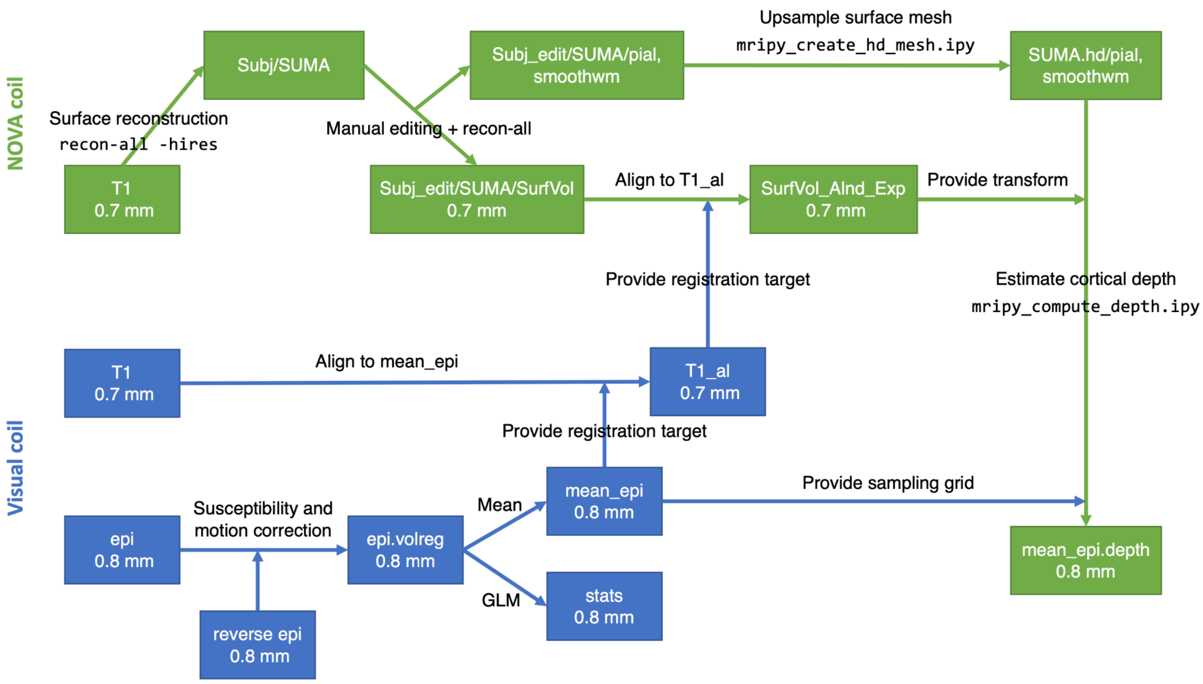

Fig. S1. Overview of the anatomical and functional MRI data processing pipeline. Blue and green boxes indicate datasets derived from the data acquired using the Visual coil and NOVA coil, respectively.

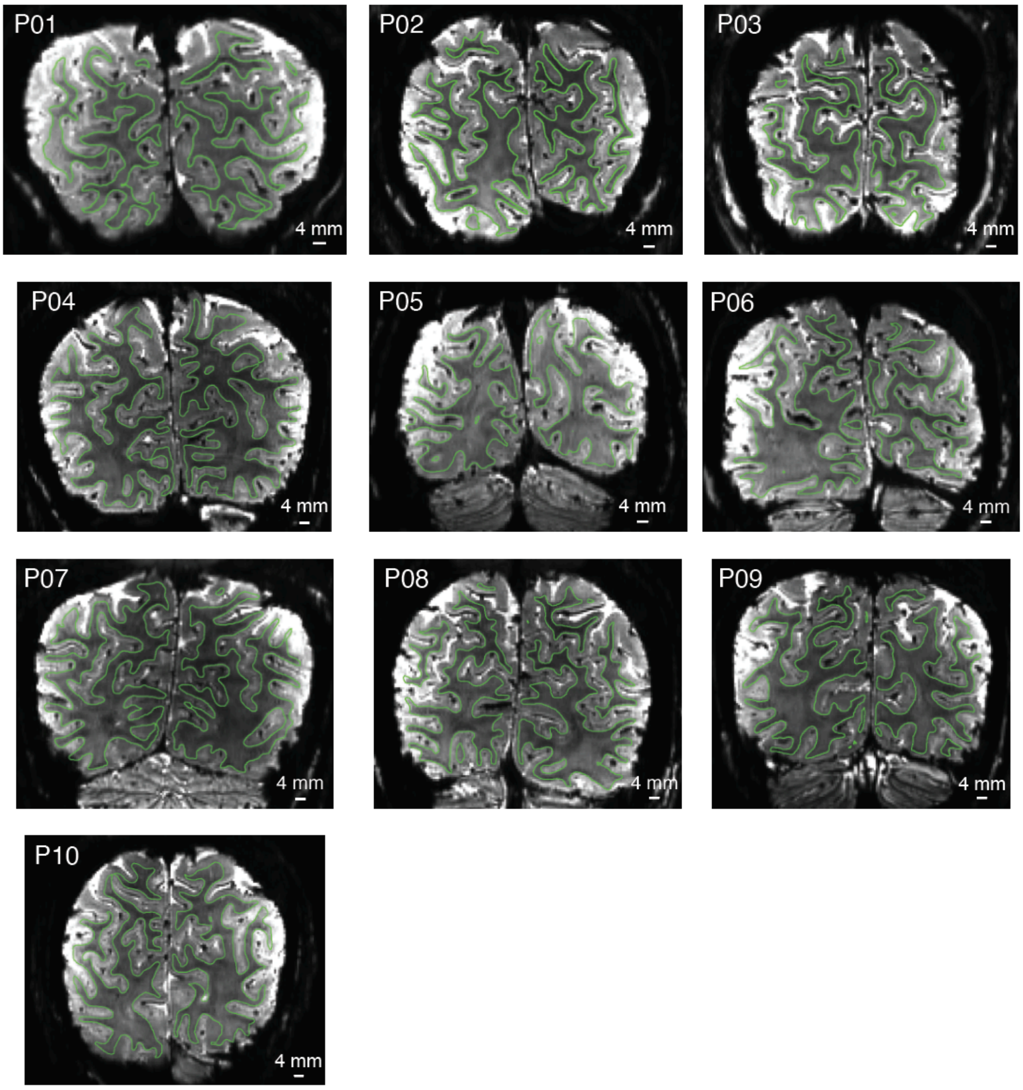

Fig. S2. Co-registration of functional and anatomical volumes. To demonstrate the registration quality of GE-EPI and T1w anatomical volumes, mean EPIs after distortion correction were shown with the white matter boundaries overlayed as solid green lines.

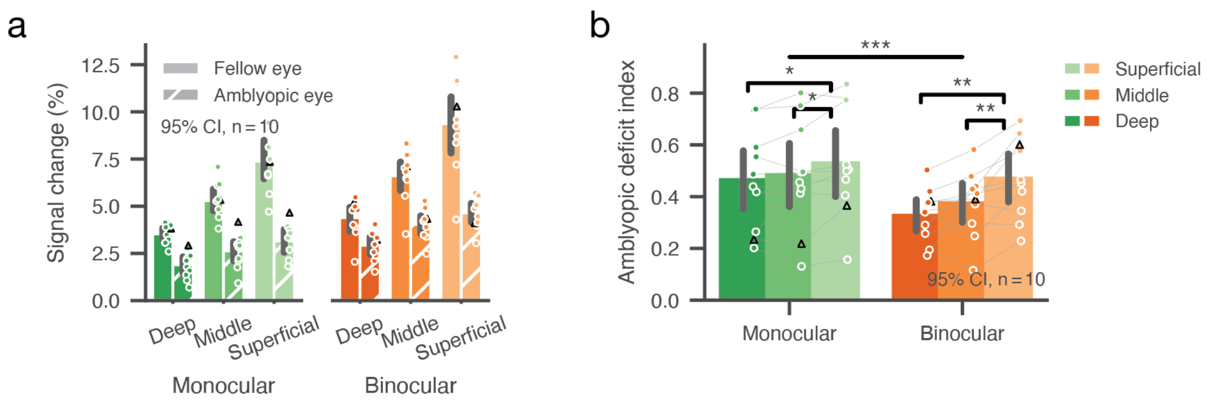

Fig. S3. Cortical depth-dependent fMRI response (a) and amblyopic deficit indices (b) in V1 for monocular and binocular conditions using a split-half approach. To compare V1 responses in monocular and binocular conditions within the same set of voxels selected using independent data, we split fMRI data into two halves (odd runs and even runs). We selected AE- and FE-biased vertices using the first half, and then evaluated the mean response with the surface ROI using the second half of the data. The superficial layers now also showed a slightly larger amblyopic deficit than the deeper layers during monocular stimulation, but the difference was more prominent during binocular stimulation as suggested by the highly significant layer-by-condition interaction. Conventions were identical as in Fig. 3a/b.

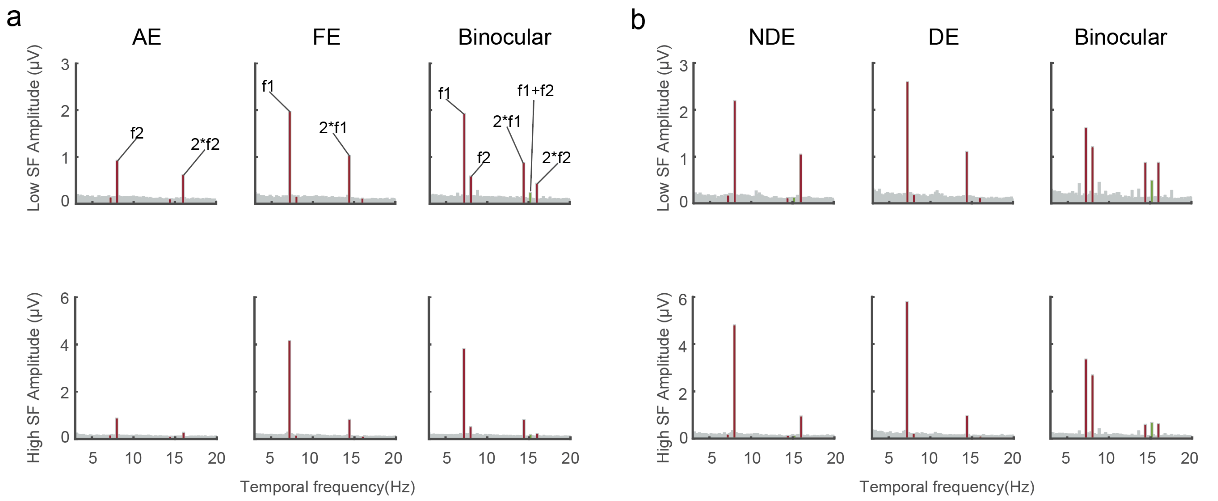

Fig. S4. (a) Group-averaged amplitude spectrums to stimuli presented to AE (f2) and FE (f1) in amblyopic subjects. **(b)** Group-averaged amplitude spectrums to stimuli presented to NDE (f2) and DE (f1) in normal controls.
